# Why do parents use packaged infant foods when starting complementary feeding? Findings from phase one of a longitudinal qualitative study

**DOI:** 10.1186/s12889-022-14637-0

**Published:** 2022-12-12

**Authors:** Anna Isaacs, Kimberley Neve, Corinna Hawkes

**Affiliations:** Centre for Food Policy, City, University of London, Northampton Square, EC1V 0HB London, UK

**Keywords:** Infant feeding, Snacking, Packaged foods, Complementary feeding, Obesity, Qualitative

## Abstract

**Background:**

The first 1000 days in a child’s life, from conception to age two, are a critical period for establishing a child’s health and development. One important element is the diet that children receive during this time. Dietary intake of infants in the UK has been shown to be high in sugar and salt, with overall energy intakes exceeding recommendations by the time they are two years of age. Commercial infant food, which forms approximately 40–60% of infants’ dietary intake, was identified in 2011 as the main contributor to sugar intake for infants aged 4–9 months in the United Kingdom. Further, evidence demonstrates inconsistencies between national recommendations on infant feeding and some of these products in terms of the type available, their nutritional value and product labelling and marketing. Given their role in infants’ diets, it is important to understand parental perceptions of these products and why they are chosen.

**Methods:**

The study comprised the first phase of an in-depth, longitudinal qualitative study which explored parents’ experiences of introducing solid foods to their infants over the first year of feeding. 62 parents/ carers were recruited to this phase when their infants were four-six months old. Data collection involved semi-structured interviews and a photo-elicitation exercise. Data from interview transcripts which focused on the purchase and use of packaged purees and commercial snacks were analysed thematically.

**Results:**

Parents/ carers drew on a range of reasons for buying both packaged purees and commercial snacks for their infants. These included anxiety over food preparation, food safety, convenience, cost effectiveness, the pull of brand eco-systems for packaged purees, and the way in which commercial snacks provide opportunities for safe development of motor skills, keep infants occupied, and allow them to take part in family rituals.

**Conclusion:**

In considering the use of packaged products as a food source for infants in public health nutrition policy, it is important to understand the broad range of factors that shape parents decisions ranging from the way that products are advertised and perceived, to the non-nutritive roles that they play.

## Background

The first 1000 days, from conception to age two, provide an important foundation for establishing a child’s health and development [1]. How a child develops during this period strongly influences health outcomes in later life [2]. One important component of health is diet: rapid weight gain in infancy (displayed as upward crossing of centiles) is associated with an increased risk of obesity in adulthood together with attendant health impacts [3, 4]. Socioeconomic differences in weight are established early. Data from 2020 shows that in the first year of primary school 13.3% of children in the most deprived areas of England (i.e. the most deprived 10% of areas according to the Index of Multiple Deprivation) were living with obesity, compared to 6.0% of those living in the least deprived areas [5]. Infant diets begin to be established when solid foods are introduced. Thus, what and how foods are introduced, for instance parent-led (purees) or baby-led (finger foods), may impact diets and health in later life [6]. Flavour experiences in the early years have been shown to affect later food preferences and practices [7], while early introduction of solid foods (before six months), which is association with socioeconomic position [8], has been linked to increased likelihood of overweight and obesity [9, 10].

Dietary guidelines for introducing solid foods to infants in the UK, termed complementary feeding, recommend that infants should start solid foods at around six months of age, and that the only drinks offered between six and 12 months should be breast milk, infant formula, and water, and that nutritious snacks should be introduced around 12 months of age [4, 11]. Parents are also encouraged to introduce a wide range of solid foods with diversification of flavours and textures increasing in stages [4]. Dietary intake of children in the UK has been shown to be high in sugar and salt, with overall energy intakes exceeding recommendations by the time they are two years of age [12]. The 2013 Diet and Nutrition Survey of 2683 Infants and Young Children reported that 75% of the children included in the survey (aged 4 to 18 months) had parent-reported intakes that exceeded the UK Estimated Average Requirement (EAR) for energy, and that the percentage exceeding the EAR increased with age following the introduction of solid foods [13]. Commercial infant food was identified in 2011 as the main contributor to sugar intake for infants aged 4–9 months in the UK [14], with commercial baby and infant foods and drinks forming part of the diet of approximately 40–60% of infants [15]. Evidence demonstrates inconsistencies between national recommendations on infant feeding and some of these products in terms of the type available, their nutritional value and product labelling and marketing. This may encourage the introduction of foods before the recommended age or frequencies and amounts of food and ingredients not recommended as part of a healthy diet for a particular age group [15, 16]. Baby snack foods (e.g., rice cakes and snacks with melting texture) have been the growth driver in the UK baby food and drink market in recent years, with approximately 65% spend increase from 2014 to 2018 and a 10.8% growth in volume sales [15]. A study in Ireland showed that a high proportion of the available snack food products targeted at young children aged 4–12 months contained high levels of energy from fat and total sugar [17]. There has also been a rise in the sugar content of savoury infant foods [18]. With a large proportion of foods marketed as healthy for children shown to fall below ideal nutritional standards [19] it is important to understand why these foods are being chosen.

A study from the United States showed that the main reason for parents to choose commercial infant foods and drinks was health, followed by taste and baby’s comfort [20]. Parents want to know what is in the foods they buy and save money where possible, regardless of income [20]. There is a trust in food manufacturers to know what is best for babies [21]. However, understanding packaging and ingredients can be made difficult by confusing health claims and messages on baby foods [19, 22]. For instance products often claim to provide one of the five recommended portions a day of fruit and vegetables; yet in a recent study, 75.4% of products making this claim were made up of less than 80 g of fruit and vegetables, which is the amount that constitutes one portion [19]. The transition to solid foods and drinking from cups goes beyond nutrition: parents view it as a sign of development and contextual factors have been shown to be more important than nutritional ideals in shaping feeding practices [20, 23]. For example, snacks have been shown to be used to manage behaviour rather than provide nutrition [24]. Ultimately, parents wish for babies to fit into family mealtimes and have healthy eating practices [25].

There is currently limited research in the UK that explores why parents choose commercial foods for their infants as they start complementary feeding, nor what role they play in infants’ diets. Understanding why parents buy commercial foods that may not match national dietary guidelines is important when considering opportunities to positively influence health outcomes in the early years and later in life. Thus, through an in-depth study into parental experiences of the infant feeding transition from milk to solid foods, we sought to understand parents’ perceptions of packaged commercial foods and their reasons for choosing them.

## Methods

The findings presented here are drawn from the first of three phases of a remote, qualitative longitudinal study that explores the feeding experience of 62 parents and carers of young infants during a one year period over the infant feeding transition. Parents and carers were interviewed when their infants were approximately 6, 12, and 18 months old. Qualitative longitudinal research allows for analysis over a period of transition as well as at specific points in time [26]. This provides a unique opportunity to understand the facilitators or challenges experienced during life course transitions such as starting solids [27]. The methods used comprised a semi-structured interview, followed by a photo-elicitation activity and follow-up interview. AI and KN collected data for the first phase between July and December 2020. Given the restrictions imposed by the coronavirus pandemic all data was collected remotely either over the phone or via video conferencing.

### Setting, sample and recruitment

Participants comprised a purposive sample of 62 parents and carers with infants aged 4–6 months at the study outset. All the infants were being cared for at home full-time during the first phase of the study. A sample size of 62 was chosen based on experiences of similar qualitative research projects. 62 was deemed large enough to allow a big enough group of parents each of high, middle and low socioeconomic position, while still remaining manageable within the boundaries of the project. Parents had either just initiated complementary feeding, or were about to start. We did not screen for, or exclude, infants who were born prematurely, nor did we recruit participants on the basis of any prior interest in food.

Approximately equal numbers of participants of high, low and middle socioeconomic position (SEP) were sought. SEP was ascertained through asking potential participants to fill out an online survey containing a series of questions validated by Kininmonth et al. to calculate SEP [28]. To assign participants to either high, medium or low SEP, we calculated the highest and lowest possible SEP scores and divided the range into three equal groups.

The primary method for recruiting participants was social media. AI and KN compiled a list of parent and baby Facebook groups across England and KN either posted a recruitment flyer or asked the administrators of the group to do so. A secondary method involved snowball sampling where participants were asked to share details of the study with friends or acquaintances. Flyers contained a link to fill out a survey which contained questions to calculate SEP as well as gather contact details. Participants were then contacted and recruited from each SEP bracket until we had roughly equal numbers from each SEP bracket. In total 62 participants were recruited: 18 low SEP, 22 medium SEP and 22 high SEP. This paper focuses specifically on the experiences of the 38 parents who were already providing packaged to their infants.

### Data collection

This paper focuses on data collected only during phase one of the study. It focuses specifically on findings relevant to packaged purees and commercial baby snacks (e.g., rusks, melting puffs and flavoured rice cakes) and on the experiences of the 38 parents who were providing packaged foods to their infants.

#### Survey

Potential participants filled out an online survey which contained both demographic questions (income, education, housing, employment, car ownership, ethnicity and postcode) and space for provision of contact details. Based on this information the researchers were able to calculate SEP using Kininmonth et al’s validated measure [28]. The researchers then contacted potential participants in each SEP bracket until we had roughly equal numbers from each SEP. Initial contact involved providing more information about the study (including the participant information sheet and an offer to answer any questions). If potential participants remained interested an interview date was set and the consent form shared, to be returned digitally.

#### Semi-structured interviews

All 62 participants were recruited when their infants were aged four-six months, and took part in a remote semi-structured interview (either by phone or video-conferencing according to preference). Interviews were intended to elicit information on experiences of and perspectives on introducing complementary foods: why participants chose the foods and feeding methods they did, and what personal, social, cultural, and economic factors shaped these decisions. These topics were discussed in the context of life with a young infant, and the impact of the COVID-19 pandemic which coincided with the infants’ births. Interviews, which lasted between 40 and 70 min, were audio recorded and transcribed verbatim.

#### Photo-elicitation

Following the first interview, participants were asked to spend a week taking photos of things that made infant feeding either harder or easier. We intentionally left the question vague, as we wanted the participants to think broadly about all the issues that feed into complementary feeding. To help provide clarity we offered some examples, such as a photo of a piece of equipment used to prepare baby food, or a particular food that is easy (or difficult to prepare). These photos were then sent to the researchers via WhatsApp or email and discussed in a second interview. Photo-elicitation is a participatory, photo-based method in which the participant takes photos related to a specific topic over a set period of time (in this case a week), and the photos and their meanings are subsequently discussed with the researcher [29]. Photo-elicitation was chosen to provide additional insight into the context in which feeding practices are formed. Since participants are able to photograph what they wish, when they wish, the participant, rather than the researcher, is able to control this element of the research narrative. Participants went through each photo, describing what it represented and why they took it. The discussions elicited by the photos were analysed, rather than the photos themselves.

### Data handling and analysis

Following transcription all interview transcripts from the semi structured interviews and photo-elicitation interviews were uploaded into the qualitative research software NVIVO 12. Analysis broadly followed Braun and Clarke’s five-stage process of reflexive thematic analysis which runs from familiarisation with the data to the eventual development and writing up of themes [30, 31]. This process was adapted, however, to accommodate for the large data set. Rather than generate a fixed codebook or coding framework and measures of inter-coder reliability, reflexive thematic analysis considers analysis to be a subjective process in which open coding is encouraged. While codes (e.g., ‘texture’ or ‘self-feeding’) may be theoretically informed, they are not generated in advance of a close reading of the transcripts [30]. Given that two researchers were needed to analyse the large amount of data, rather than one which is more traditional for reflexive thematic analysis, it was necessary to develop a loose coding framework. This still allowed for the addition of new codes as the process progressed. In the initial stage both AI and KN read and open-coded three interview transcripts and three photo-elicitation transcripts (one participant each of high, low, and middle SEP). They then discussed these open codes and developed a coding framework. AI and KN then coded half of the transcripts each, adding new codes where relevant and discussing the generation of new codse. Finally AI and KN used the coded data to develop explanatory themes regarding the introduction of complementary foods. The themes explored here are those specifically relating to use of packaged infant foods, both packaged purees and infant ‘snacks’ ,e.g., rusks, melting puffs, flavoured rice cakes.

During the process of analysis both researchers reflected on their own experiences as women without children, interrogating their assumptions about ‘healthy’ and ‘unhealthy’ feeding practices. They noted these reflections down and shared them with each other during the analysis phase. While all research findings are to some extent shaped by the researchers, this ensured that they maintained an awareness of potential biases and their impact on the analysis throughout.

### Ethics

Ethics approval was sought and obtained from [ethics committee name redacted for anonymity] and the project was carried out according to the rules and regulations set out by the committee. Informed consent was obtained from all participants, including for publication and sharing of the anonymised data. Decisions around feeding infants can be associated with considerable guilt. AI and KN, who have extensive experience of working with a diverse range of participant groups, sought to maintain the interviews as a non-judgemental space, where they sought to learn from, rather than question participants’ experiences. The researchers did not probe as to why participants chose to breast or bottlefeed (unless initiated by the participant) as this is a particularly sensitive area and not a primary research aim.

Any identifying features have been removed and all names in the findings are pseudonyms.

## Results

### Participants

In total 62 participants (18 low SEP, 22 middle SEP, 22 high SEP) took part in a semi-structured interview, with 60 also completing the photo elicitation exercise. Detailed participant information can be seen in Table 1. All participants had an infant aged between four and six months when recruited. While the majority had introduced at least some solid food, 10 participants had not yet done so and so the interview focused more on their plans and intentions. Of the 52 who had started solids, 14 had not yet given any packaged foods. 38 participants were currently providing packaged foods (‘snacks’and/or purees) either sometimes or regularly. While the types of first foods introduced varied across SEP (e.g. baby rice vs. vegetables), we did not note any particular differences in provision of packaged foods.

**Table 1:** Participant demographics

Demographic Information	
**Number of participants**	
	62
**SEP**	
Low	18
Medium	22
High	22
**Gender of parent**	
Male	1
Female	61
**Ethnicity**	
White British	43
White	5
British	9
Irish	1
Indian	1
Black Caribbean	1
Norwegian and Greek	1
South Asian	1
**1st child**	
Yes	28
No	34
**Single parent family**	
Yes	4
No	58
**Use of packaged foods/snacks**	
Not started solids yet	10
None (yet) - but started solids	14
Sometimes	30
Frequently	8

### Key findings

This section describes our findings related to the use of (a) packaged purees (primarily used as main meals) and (b) commercial snack foods. As noted, while there were some differences across SEP in terms of which foods were introduced to infants first (e.g., baby rice and porridges were more common as a first food amongst low SEP families), we did not record significant differences in approaches towards packaged foods. We therefore focus here on the commonalities of experience, and reflect on this in the limitations. We explore first key reasons for choosing packaged purees: safety, reassurance and cost effectiveness, in addition to convenience when out and about. Second we consider why participants provided packaged / commercial snacks, including absence of ingredients that were considered unhealthy, their role in aiding motor development, keeping infants occupied, and involving them in family rituals such as teas or birthday celebrations.

#### Packaged purees provide reassurance about health, suitability and value for money, in addition to being a convenient option

Not everybody in the study used packaged purees, but many had, or were considering starting to use them. These participants reflected on occasions where they would use purees such as when on holiday or out and about. In addition to the convenience of a pre-made product, participants who offered purees regularly drew on a range of other reasons for using them:

##### Purees feel safe for parents who are anxious about feeding and packaging reinforces this

Participants who felt concerned either about their own cooking skills or about being able to prepare something that was suitable for an infant were able to rely on packaged purees as a food source that was considered totally safe for infants. Participants who chose them felt that purees could be relied on to have the correct levels of salt and sugar, be an appropriate texture, and avoid ingredients that infants should not consume.

“It’s mostly shop-bought, so mostly pouches and jars of baby food. He will try stuff off our plate. I don’t know, I think I’m just worried about if I’ve added salt to something, or is there too much sugar in it? At least I know with the actual baby food it’s completely suitable for him” – Jeni, high SEP.

“I have been doing it a little bit here and there every couple of days. Ella’s Kitchen pouches, I’ve been trying him with because I can see all the ingredients. I don’t have the confidence or the time, really, to make stuff myself, and we don’t have the storage in my freezer either to bulk make and store, so the Ella’s Kitchen pouches are just easy in the cupboard.” Felicity, low SEP.

The age ranges listed on products provided an additional layer of security that a specific product was suitable. Participants highlighted that certain products were stated suitable for four-six months, despite UK guidelines recommending around six months as the optimum time to introduce solids. This gave some the reassurance that they could introduce solids at an earlier age.

“A friend of mine, neighbour with two children, mentioned porridge and baby rice, so I thought, oh. And it said four to six months plus, so I thought, oh, that’s perfect. What you mentioned about age is probably what I got drawn to on the packaging with the Aptamil, because it said four to six months plus. So I was, like, oh right, she’s five months, I can start.” – Alisha, middle SEP.

**Fig. 1:**
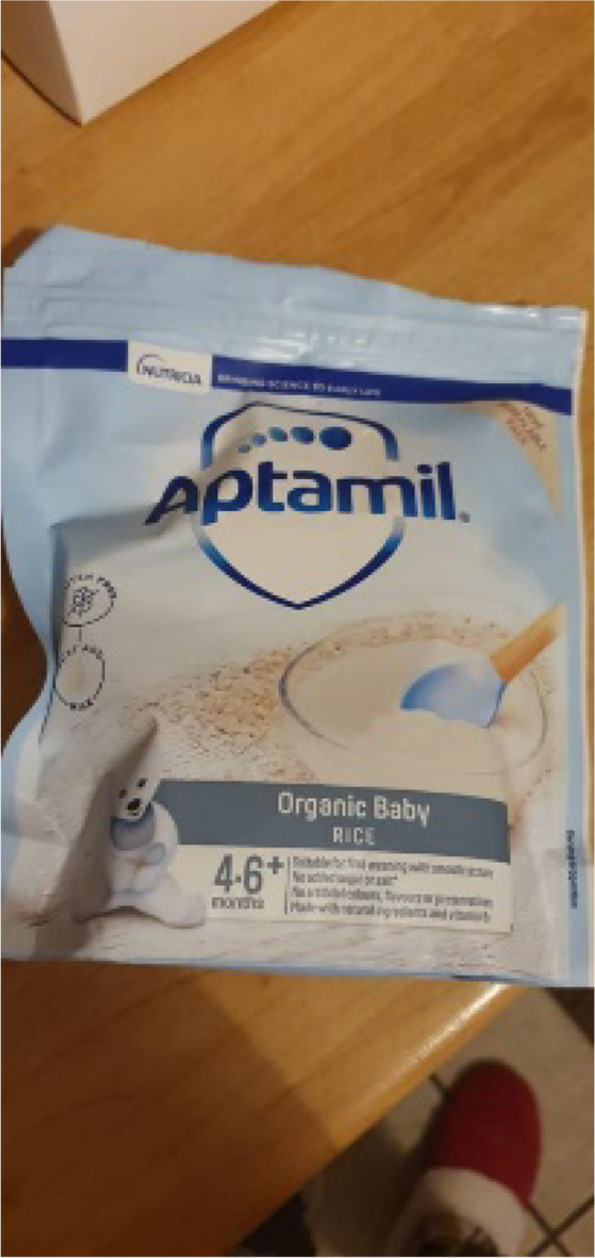
Aptamil baby rice, suitable from 4 months

“Some of the packaging still says four plus months [Fig. 1] which obviously does contradict the NHS advice, so that’s probably a bit confusing for people if they weren’t somebody who would go and look at that information or research for themselves. That’s probably a bit contradictory, yes. I think probably if I hadn’t read that and hadn’t followed people weaning their children online on Instagram, I probably would have looked at that packaging myself and thought oh, I’ll start weaning at four months and not necessarily know that that wouldn’t have been the best time for my baby.” – Julia, high SEP.

##### Brand eco-systems provide reassurance

**Fig. 2:**
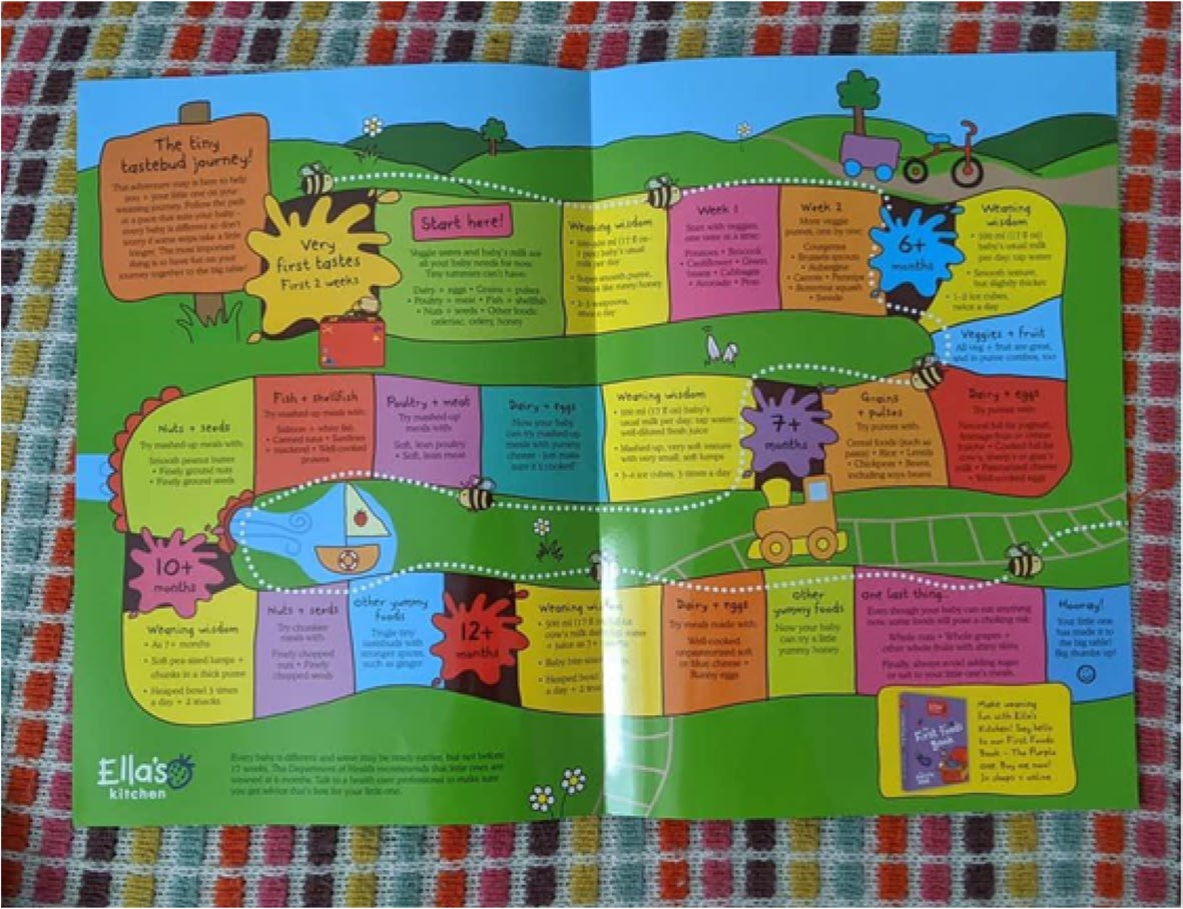
Snakes & Ladders game in Ella’s Kitchen weaning pack

The ‘eco-system’ around certain brands (e.g., marketing and advertising that is broader than the products themselves), helped instil a sense that they were not only safe and appropriate but a natural part of the journey of introducing solids. Most participants, for example signed up for a free weaning pack from a key baby food brand [Fig. 2]. This provided clear information about a process which was often daunting, as well as vouchers for free product samples. Information about these packs was often gleaned from social media, included in promotional packs given out in hospital, or filtered through parent networks.

“This [Brand weaning pack] came in the post. I did it online, I saw somebody posted [it] on one of the mum’s apps, that you can get this for free, just to get some ideas on what to feed him. I like the snakes and ladders kind of thing, you could tell the different types of foods and stuff. It also gives you a timeline, with ages as well” – Kayleigh, middle SEP.

“Ella’s Kitchen is designed for first time mums if you like, because everything on their packaging is self- explanatory. And it tells you although this is a first finger food remember you’ve got to supervise them, you’ve got to brush their teeth, and you’ve got to do this” – Amy, low SEP.

**Fig. 3:**
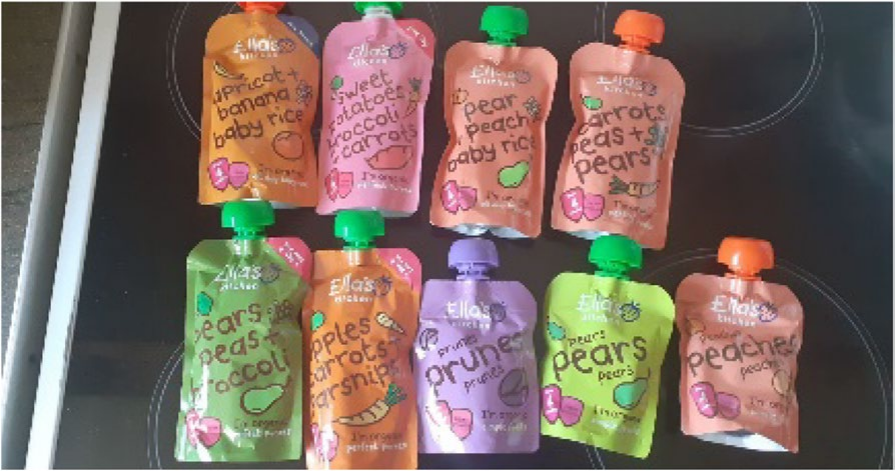
Assortment of Ella’s Kitchen pouches

Trust, inspired by the advertising eco-system was then reinforced by the descriptions on the front of packages. Packaging from Ella’s Kitchen (and other brands) were considered simple, honest and devoid of jargon [Fig. 3], reassuring parents that there were no “nasties” or unwanted ingredients.

“Basically they’re [Ella’s are] easy to read ingredients wise. It is exactly what it says on the packet. There is no added salt, sugar, sweeteners. And obviously with me having to be careful with what I’m feeding [baby] they’re just very clear-cut and there’s no nonsense, and there are no scientific big words to try and decipher.” Alice, low SEP (infant also had a dairy allergy).

The ways in which ingredients were presented on the packaging also contributed to this sense of security. Rather than scrutinising the detailed ingredients on the back of packages, participants tended to look at what was listed on the front, particularly if it was a brand they had purchased before. Descriptions that emphasised the simplicity of the product and the absence of unwanted ingredients generated confidence.

“So I look more at the front of a packet rather than at the back. So I probably for the baby products I don’t look at the ingredients itself but I would look at the front for that to tell me what they contain” – Julia, high SEP.

##### Purees were often considered to be a cost effective option

While purees were not necessarily cheaper than raw ingredients in actuality, a number of participants considered them a cost effective option, even when on a low income. Participants who preferred purees for this reason felt that they wasted less food this way, since if their infant rejected a particular flavour it was only one jar rather than a whole batch. This was the case especially for those who were eating different foods to their infants.

“I started to buy because in the beginning I was just making food and wasted to the bin because he wasn’t eating. I ended up buying ingredients, I had to look at ingredients because he was not eating so I just tried to start to buy different flavours to see which one he likes more I was just wasting time and food like that. At least the portions he has are small and not a big batch and then he can try, see what he likes.” – Ayanna, middle SEP.

“I did want to do the fresh stuff, but I found that with him not wanting it, it’s me wasting food, and then that’s wasting money. So, I thought, well, if I bought a jar and he doesn’t like it, then technically it’s only a jar at the end of it, not like a bag of carrots or a bag of sweet potato or a bag of broccoli. – Felicity, low SEP.

#### Packaged snacks are not chosen for nutritional content or to fill baby up, but to fulfil non-food requirements

**Fig. 4:**
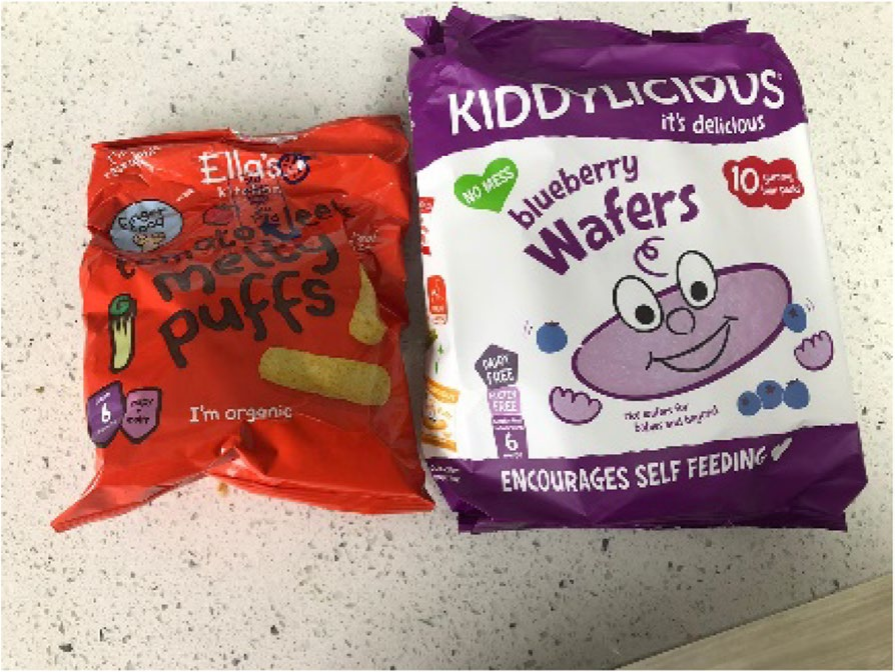
Selection of snacks with melting textures

Provision of packaged infant ‘snacks’ including flavoured rice cakes, fruit and vegetable flavoured ‘puffs’ and, less commonly rusks, was very common amongst the participants. These tended to be offered both just before or after a ‘main’ meal, or at a different point during the day as a snack. Participants’ explanations of why and when snacks were provided demonstrated that they were not considered food in the sense of providing nourishment or calories, but rather were offered for a range of other reasons. As a result, there was less concern about the positive nutritional content of the snacks [Fig. 4], and rather assurance was sought that they were not actively harmful for an infant. Participants commented on the absence of key ‘bad’ ingredients, such as sugar and salt in the products they chose.

“Well they’ve got a few additives but they’re quite low in salt. She’ll only eat one or two, and the whole bag’s got quite low salt and they’re made of peas or whatever “- Clare, middle SEP.

“Had a look at the ingredients and they don’t have sugars and so on, it was all like rice flour and the pure fruits, so on. So I decided to give her a go, and she loves them” – Abigail, high SEP.

##### Snacks safely aid infant development

**Fig. 5:**
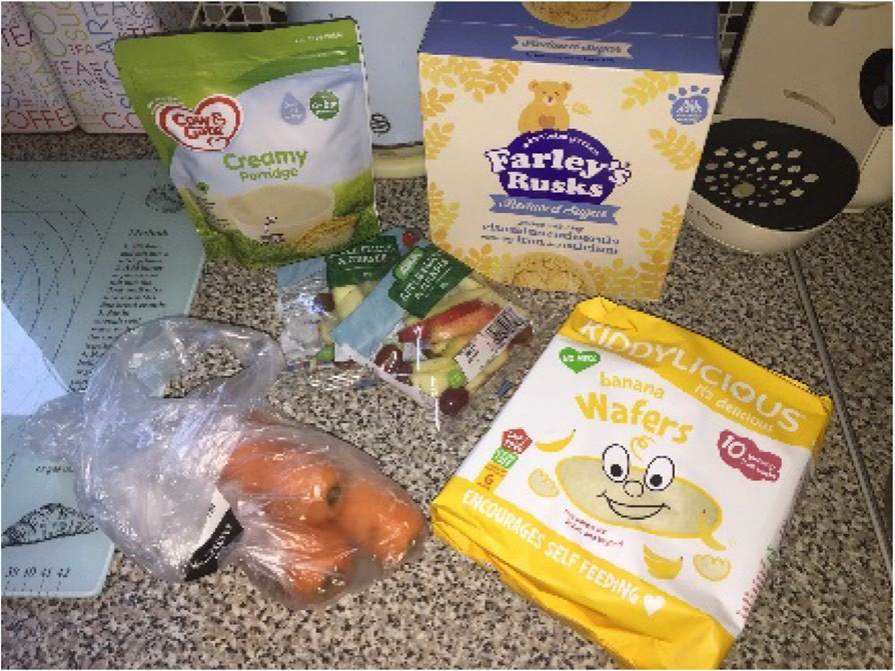
Selection of complementary foods

Many participants explained that packaged infant snacks [Fig. 5] were a useful way to assist infants with developing fine motor skills and independent eating, in a safe and controlled manner, with limited mess. Babies were able to pick up and consume snack foods themselves, but because of their soft and/or melting textures, parents felt confident that there was no choking risk. Snacks that advertised developmental benefits were therefore particularly popular.

“So it’s a pureed meal to get the nutrients in and then the [packaged] finger foods for him just to play with and explore the textures in his mouth” – Amy, low SEP.

“The Organix ones, the carrot crisps, I tend to give quite early on, because they sort of melt in the mouth. They’re quite nice for them. And they can actually hold those in their hands, as well” – Melissa, high SEP.

“They’re [rusks are] quite good, actually, they go all mushy in his mouth, just so that he can still learn how to feed himself as well as me feeding, because that’s what he prefers” – Leanne, low SEP.

##### Snacks keep infants busy

**Fig. 6:**
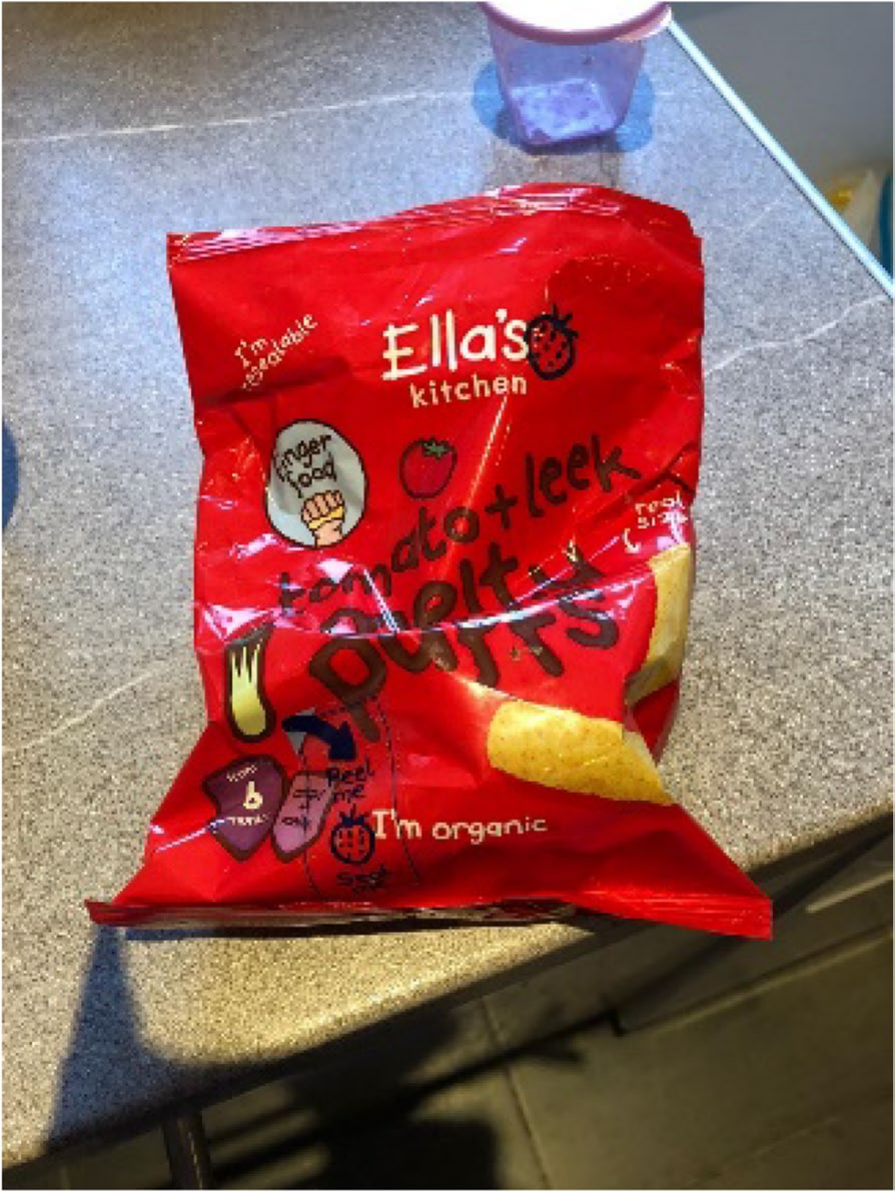
Savoury snack with a melting texture

Snacks also played a useful role in keeping infants occupied and satisfied if parents were doing chores or trying to eat themselves. Packaged snacks Fig. 6] [were extremely popular with the infants and so were able to hold their attention for long enough that parents could finish what they were doing.

“So, they’ve got these Kiddylicious wafers. She adores them. Normally she’ll have her dinner, and then she gets one of those while we’re eating our dinner, so I can eat my dinner in peace” – Phoebe, low SEP.

“And sometimes if he’s getting a bit whingy, it’ll distract him a little bit. So he does have a few, not crazy amounts. I just give him a few a day out of the packet. Some days, he doesn’t have any. Or if we’re out shopping and he doesn’t want to be in his pram, I’ll give him some then. But it helps him because he feeds himself with them”. Philippa, middle SEP.

##### Snacks offer a way to integrate infants into family rituals

Finally, snacks provided a way for infants to be part of wider family rituals, such as having a treat or a piece of cake. Many participants didn’t feel it appropriate to give their infant the same treat as the rest of the family but still wanted a way for the infant to be involved either in a special occasion such as a birthday, or simply be part of a regular meal.

“The main reason she had them yesterday is because we went for a meal, so she sat at the table with us whilst we were having a meal, just eating her little snack sticks so she felt part of the table as such” – Abigail, high SEP.

**Fig. 7:**
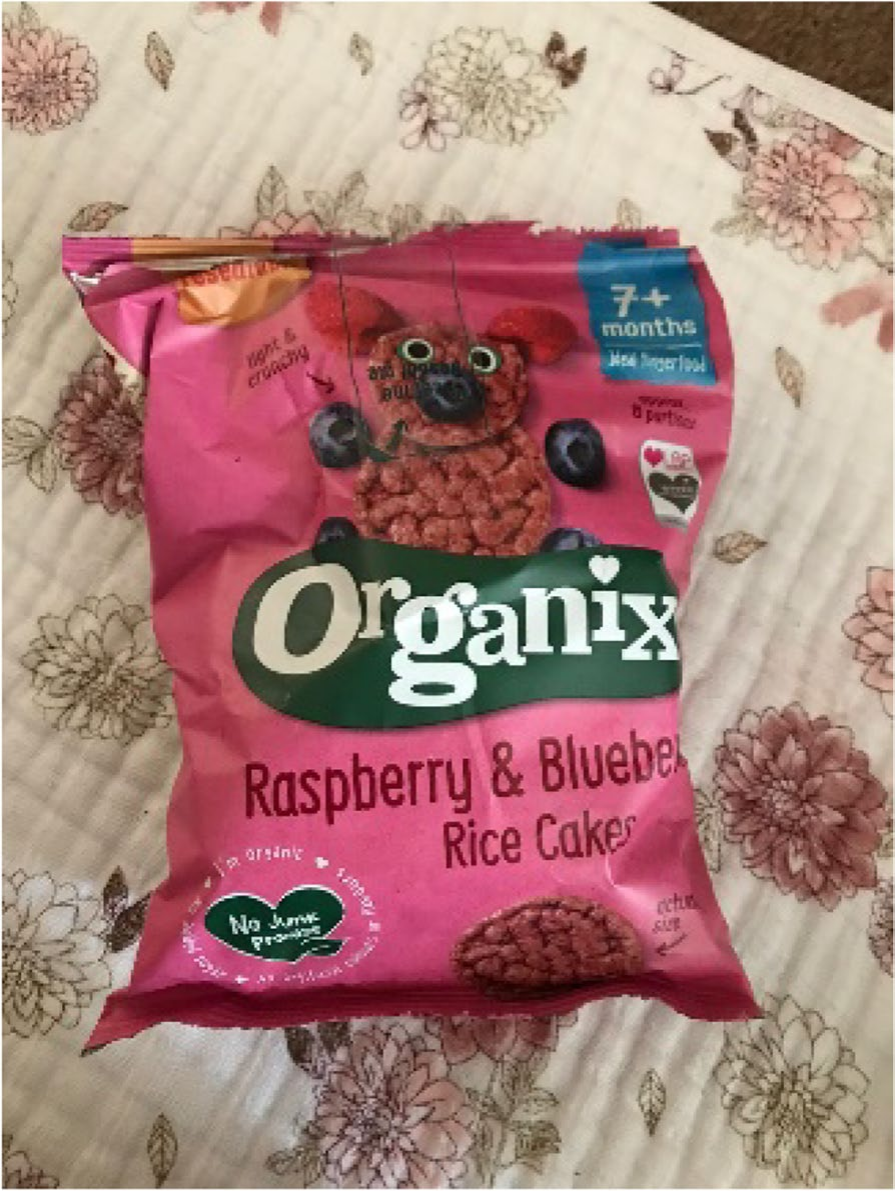
Fruit-flavoured rice cake used as a treat

“My mother in law brought those [rice cakes, Fig. 7], because we were having a family gathering and she wanted to, she brought us all cake, and wanted to give [baby] something - Zoe, high SEP.

## Discussion

This study explored why parents/carers of four-six month old infants across a range of SEPs chose packaged foods (both purees and commercial snack foods), and what their perceptions of those foods were. This is important to understand (a) parents’ perceptions of products that may not fall in line with nutritional targets (b) the role that food companies play in reinforcing certain perceptions and (c) the broader needs and concerns parents have that shape their purchasing practices.

Packaged purees were chosen because, in addition to convenience when out and about, they provided reassurance to parents who were anxious about what to feed infants, were considered cost effective, and because parents felt reassured by the advertising and packaging associated with certain brands. Some parents, whose infants had allergies, were more confident giving packaged foods because they could easily avoid allergens. Indeed a study from Australia confirmed the limited presence of allergens in packaged infant foods [32].

Previous research on infant feeding has noted that packaged purees were chosen by parents because they were convenient, tasty, perceived as healthy and (for some parents) considered safer [20, 33]. In addition to these factors, our findings demonstrate the various ways the products are able to quell parental anxiety and offer parents confidence, as well as how branding and packaging played into these concerns and provided reassurance. Finally the finding that parents, even on a low income, found purees cost effective is important in helping understand the appeal of products that might seem more expensive on the surface.

Commercial snack foods occupied a different space in parents’ minds to purees. Snacking (of both commercial and homemade foods) was a common practice across SEP when starting solid foods, reflecting previous research in the US [34]. While parents still wanted their infants to have healthy diets overall, commercial snacks were not chosen for their nutritional profile or contribution to infant diets, but to fulfil other functions, namely keeping infants occupied, assisting with motor skill development (without risk associated with other finger foods that did not melt in the mouth) and allowing children to take part in wider family rituals. In line with a desire to promote motor development, but avoid choking risks, parents often chose snack products that both highlighted their developmental properties but also emphasised soft and melting textures. Qualitative research by Moore et al. similarly found that anxiety around choking and gagging led parents to choose packaged snack foods that dissolved easily [35].

Foods carry meaning beyond nutrition and sustenance, to fulfil numerous other roles. Because commercial snacks were not considered food in the same way as other packaged products, parents looked for different proxies to ensure that they were appropriate for their children. It was less important that they were explicitly healthy, or contributed to infants’ overall diets, but rather that they were not actively unhealthy or unsafe.

For both purees and commercial snack foods, descriptions on the packaging helped to reinforce parents’ notions that these were appropriate products. For purees, front of pack labeling that provided simple ingredients lists showed parents that the products were pure and nutritious.This was further reinforced by terms such as ‘organic’ and ‘natural’ as well as age recommendations that confirmed suitability for a certain age. Research has shown however that front of pack ingredients lists do not always accurately reflect what is actually in the product [15]. Furthermore, age recommendations do not always reflect current guidelines that foods should only be introduced from around six months [15] and have been found to encourage early weaning [36]. For snacks, descriptions that highlighted the absence of added salt or sugar or that contained phrases such as ‘encourages self feeding’ reassured parents both that these products were safe and that they had particular non-nutritive benefits. Again front of pack labelling does not always reflect the reality of the products with many fruit-based snacks being high in free sugars even if these are not added [15, 17, 18, 37]. For some brands, cues on packaging were also received within a context where wider brand presence created a sense of trust and familiarity.

## Limitations

There were a number of limitations of this study. First although we sought to recruit a soioeconomically diverse sample and were largely successful in doing this, we recruited very few participants who were actively or openly concerned about their finances. This may have explained the limited variation in approaches to packaged foods between each SEP bracket. Second, our participant sample was relatively ethnically homogenous and so the voices of those from migrant and minority backgrounds are limited.

## Conclusion

In order to consider how public health nutrition policies can help support parents in an infant’s first 1000 days, it is critical to understand how packaged foods that may not fit nutritional ideals are perceived, and the roles they play in the lives of parents with infants. Parents chose packaged purees and commercial snack foods for a wide range of reasons from safety, to cost effectiveness, to reasons that have a less direct relationship to food and nutrition such as keeping infants occupied and integrating them into family rituals. Parents’ concerns and priorities were reinforced by product packaging that suggested products were safe, appropriate and pure and by products that did indeed meet many of their needs. Understanding the needs of parents that extend beyond nutritional composition is critical in ensuring that they have options that both meet their priorities and are nutritionally appropriate for infants.

## Data Availability

The datasets used and analysed for this study have been deposited with the UK data service and is available at: https://reshare.ukdataservice.ac.uk/855491/.

## Abbreviations

DNSIYC: Diet and Nutrition Survey of Infants and Young Children
EAR: Estimated Average Requirements
SEP: Socio-economic Profile
